# A giant aneurysmal bone cyst of the rib: Case report

**DOI:** 10.3892/ol.2013.1642

**Published:** 2013-10-25

**Authors:** JUNTANG GUO, CHAOYANG LIANG

**Affiliations:** Department of Thoracic Surgery, Chinese People’s Liberation Army General Hospital, Beijing 100853, P.R. China

**Keywords:** aneurysmal bone cyst, rib, tumor

## Abstract

An aneurysmal bone cyst (ABC) is a benign tumor of the skeletal system, which most frequently occurs in long bones. An ABC arising from the rib is extremely rare and it is difficult to distinguish from other types of rib tumors. The present study describes an unusual case of a large ABC in the rib of a 17-year-old male. The entity is discussed with particular emphasis on the clinicopathological features, differential diagnosis and treatment. Due to difficulties in the pre-operative diagnosis, a possible diagnosis of ABC should be made aware when confronting an expansile rib mass. An en bloc resection of the mass and the affected portion of the rib is mandatory to obtain a satisfactory outcome.

## Introduction

Aneurysmal bone cyst (ABC) was first reported by Jaffe and Lichtenstein in 1942 (1). The term was used to describe the ‘blow out’ radiographic appearance and blood filled contents of the cystic spaces (2). In pathology, ABCs are destructive, expansile bone lesions characterized by a reactive proliferation of connective tissue containing multiple blood-filled cavities. Presumably due to the local hemodynamic disturbances, the process arises de novo in bone or is engrafted on pre-existing bone lesions histologically identifiable in 30% of cases (3). ABC occurs predominantly in children and young adults, mainly involving the long bones and the vertebrae (4). The rib is a rare location for ABC. The size of the majority of cases that have been reported in the literature is <10 cm. The present study describes the case of a huge ABC of the rib presenting in a 17-year-old male, which was successfully treated by surgical resection. Written informed consent was obtained from the patient.

## Case report

A 17-year-old male was admitted to the Chinese People’s Liberation Army (PLA) General Hospital (Beijing, China) with a chief complaint of progressive right-sided chest pain and chest distress for two months. There was no history of trauma, fever or respiratory embarrassment accompanying the pain and mass. On physical examination, the right posterior chest wall in the region of the seventh rib bulged to a minimal degree, but it was not tender to palpation. The respiratory sounds were slightly decreased on the right side.

**Figure 1:**
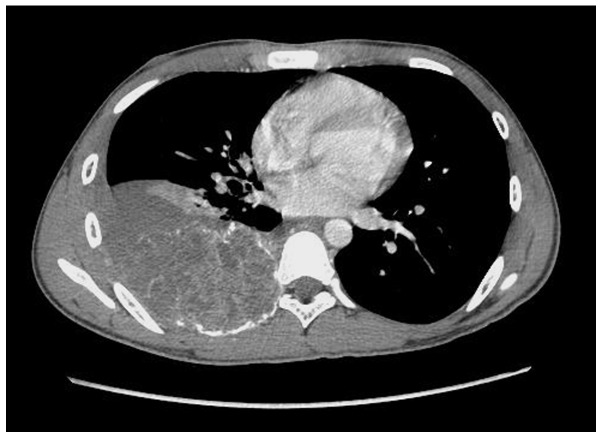
Computed tomography scan showing an irregularly shaped 12×10×10-cm expansion mass beside the 7th thoracic vertebra. Disappearance of the cortex and lytic changes in the 7th ribs are demonstrated. Atelectasis of the right lower lobe is also evident.

Plain and lateral chest radiographs revealed a posterior chest mass on the right side. Computed tomography (CT) of the chest revealed an irregular-shaped 12×10×10-cm tumor beside the 7th thoracic vertebra, which occupied almost half of the chest cavity. A mottled calcification was observed in the core of tumor. The tumor was mildly enhanced in the contrasted scan. The mass had expanded and destroyed the posterior part of 7th rib. Atelectasis of the right lower lobe and pleural effusion were also observed in the CT (Fig. 1).

CT-guided aspiration biopsy pre-operatively demonstrated that a neoplasm of spindle cell, solitary fibrous tumor of the pleura was possible. As the CT findings suggested the possibility of a malignant lesion, including sarcoma, surgery was performed on June 3, 2011.

**Figure 2:**
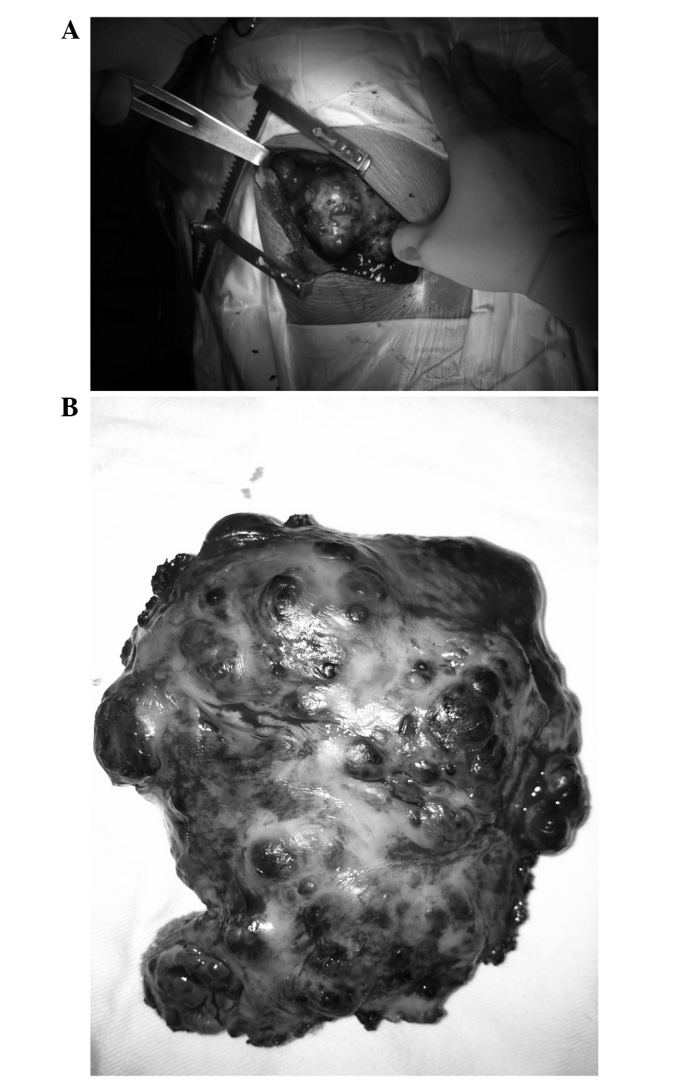
(A) Profile of the aneurysmal bone cyst, which originated from the 7th rib, during surgery. (B) Macroscopic appearance of the resected specimen.

A right-sided thoracotomy revealed a 12×10-cm mass that originated from the posterior half of the 7th rib, which was medium-hard in texture. Numerous blood-filled cysts protruded from the surface of the mass, and an abundant blood supply was observed in the margin of the tumor (Fig. 2). The main vessels leading into the mass were sutured. Due to a broad-base pedicle and poor exposure, the mass was excised totally en bloc with the affected part of the 7th rib first. Other adjacent parts of the rib, including the periosteum and adjacent intercostal muscles, were subsequently resected.

**Figure 3:**
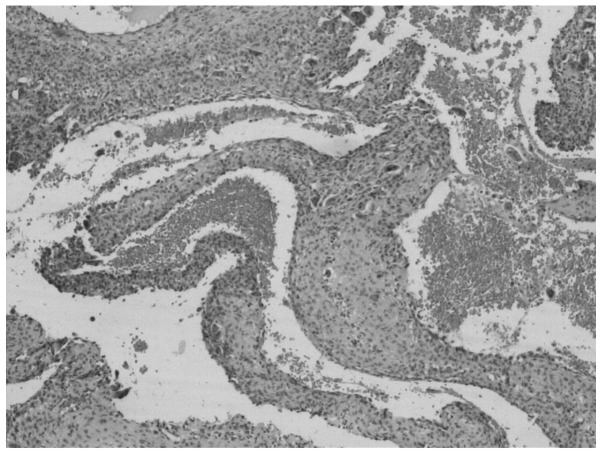
Hemorrhagic areas within a cystic space (hematoxylin and eosin staining; magnification, ×400).

The microscopic examination demonstrated multiple blood-filled cavernous spaces that were separated by fibrous connective tissue, with hemosiderin pigment-laden macrophages, multinucleated osteoclastic giant cells and well-developed vascular spaces with hemorrhages in the septa lining the cystic spaces. There were focal areas of calcification and osteoid. The surgical margin was free. All these pathological findings were consistent with those of an ABC (Fig. 3).

The patient was discharged following an uneventful 12-day postoperative period, and has been followed up for two years with no evidence of recurrence.

## Discussion

Primary rib neoplasms comprise 5–7% of all primary bone tumors (5). ABC was first described by Jaffe and Liechtenstein in 1942 (1), and accounts for only 1.3% of all bone tumors. Therefore, ABC originating from the rib is particularly rare. An ABC may occur in every rib with the exception of the lower three (6). Although ABC predominantly occurs in people aged <30 years old and equally between genders, ABC of the rib is observed at an average age of 22.8 years and is marginally more frequent in females.

The etiology of ABC is unclear. Certain studies have considered the condition to be secondary to an increased circulatory venous pressure or trauma, causing bone absorption and blood-filled cyst formation, thus explaining the expansile nature of ABC (3). Alternatively, it is proposed that ABC may be secondary to other pre-existing bone diseases, including fibrous dysplasia, giant cell tumor and non-ossifying fibroma, and is called ‘secondary ABC’ (7). Approximately one-third of all cases are the secondary type.

The most common symptoms in a patient with ABC of the rib are chest pain, swelling of the chest wall, dyspnea, paraplegia and pathological fractures (6). Some patients with ABC of the rib have an asymptomatic clinical course. These patients may be diagnosed incidentally by chest X ray or CT. Infants with ABC of the rib may also present with respiratory distress.

Although the radiological appearance of ABC of the rib is not sufficiently specific to establish a definitive diagnosis, a CT scan is of great value to demonstrate the characteristic findings of the disease. CT may aid in showing a destructive pattern of rib lesions with a ‘soap bubble’ or ‘honeycomb’ appearance (2,8). The differential diagnosis of ABC may include Ewing sarcoma and eosinophilic granuloma, among others (9). Core needle biopsy specimens have been used to confirm the diagnosis, and their careful performance is recommended as they may increase the risk of bleeding due to the abundant blood cysts inside the ABC (10).

Grossly, the lesion is a cavity separated by septa. The spaces usually contain blood or serum. The macroscopic pathological findings tend to show a paper-thin cortex that is usually intact unless there has been a complicated pathological fracture. Under this shell, the bone is completely destroyed and replaced by various sizes of cysts containing blood or serum. In the present study, the cysts were separated by septa composed of loosely arranged spindle cells and benign giant cells. Cuboidal appearing cells line the cysts, and beneath this layer, osteoid tissue with osteoblasts are observed. Focal calcifications may also be evident (3,11).

Regarding the treatment of ABC, various methods have been described, including surgery, radiotherapy, embolization, cryotherapy, sclerotherapy and a wait-and-see strategy. To date, the ideal treatment option is total excision, as it has the lowest risk of local recurrence (12). An incomplete removal may result in the persistence and growth of the lesion. In addition, it is often difficult to obtain a precise pre-operative diagnosis. Therefore, surgical treatment is recommended, and complete local excision should be performed if possible. Curettage of the lesion often results in recurrence (11,13).

Radiotherapy should be considered only for inoperable cases. Extreme care should be taken with regard to the use of irradiation, as there is the possibility of a late development of sarcoma (14). Selective arterial embolization (SAE) has been reported as an effective treatment for ABC and may be considered in lesions whose site (spine or pelvis) or size make other types of treatment difficult or hazardous. Certain studies have suggested that embolization should be the first option for ABC treatment, as it has the best cost-to-benefit ratio (12,15,16). However, due to the lack of large feeding arteries to be embolized in the ABC reported in the present case and the requirement for repeated procedures as the sole therapy, embolization ought not to be the standard therapy or a sole therapy. Pre-operative embolization aids in controlling bleeding and minimizing the extent of surgery that is required (17). If pre-operative embolization had been performed in this case, the procedure would have been smoother with less blood loss. SAE may be a significant choice of treatment for certain ABCs and is a less invasive, lower cost and simpler procedure that is easily repeatable.

Dubois *et al* recommended sclerotherapy for ABCs to avoid surgical risk and prolonged hospitalization, when the clinical presentation and radiological appearances are typical (18). The advantage of sclerotherapy is that it is a minimally invasive, safer procedure. Repetitive sclerotherapy using polidocanol or ethibloc injection makes a considerable contribution to the therapeutic solution in certain cases in which operative treatment is extremely hazardous (19,20).

ABC arising from the rib is a rare, benign entity, which should be considered in the differential diagnosis of chest wall tumors. Complete surgical excision offers the best choice of treatment for curing ABC.
